# Biological Effects of Polyrotaxane Surfaces on Cellular Responses of Fibroblast, Preosteoblast and Preadipocyte Cell Lines

**DOI:** 10.3390/polym12040924

**Published:** 2020-04-16

**Authors:** Hiroki Masuda, Yoshinori Arisaka, Ruriko Sekiya-Aoyama, Tetsuya Yoda, Nobuhiko Yui

**Affiliations:** 1 Department of Maxillofacial Surgery, Graduate School of Medical and Dental Sciences, Tokyo Medical and Dental University (TMDU), 1-5-45 Yushima, Bunkyo, Tokyo 113-8549, Japan; masumfs@tmd.ac.jp (H.M.); yoda.mfs@tmd.ac.jp (T.Y.); 2 Department of Organic Biomaterials, Institute of Biomaterials and Bioengineering, Tokyo Medical and Dental University (TMDU), 2-3-10 Kanda-Surugadai, Chiyoda, Tokyo 101-0062, Japan; arisaka.org@tmd.ac.jp (Y.A.); sekiya.org@tmd.ac.jp (R.S.-A.)

**Keywords:** polyrotaxane, integrin, cellular adhesion, focal adhesion, proliferation, differentiation

## Abstract

Biointerfaces based on polyrotaxane (PRX), consisting of α-cyclodextrins (α-CDs) threaded on a poly(ethylene glycol) (PEG) chain, are promising functionalized platforms for culturing cells. PRXs are characterized by the molecular mobility of constituent molecules where the threading α-CDs can move and rotate along the PEG chain. Taking advantage of this mobility, we have previously succeeded in demonstrating the regulation of cellular responses, such as cellular adhesion, proliferation, and differentiation. In the present study, we investigated differences in the cellular responses to PRX surfaces versus commercially available tissue culture polystyrene (TCPS) surfaces using fibroblasts, preosteoblasts, and preadipocytes. PRX surfaces were found to more significantly promote cellular proliferation than the TCPS surfaces, regardless of the cell type. To identify the signaling pathways involved in the activation of cellular proliferation, a DNA microarray analysis was performed. PRX surfaces showed a significant increase in the integrin-mediated cell adhesion and focal adhesion pathways. Furthermore, PRX surfaces also promoted osteoblast differentiation more than TCPS. These results suggest that structural features of PRX surfaces act as mechanical cues to dominate cellular proliferation and differentiation.

## 1. Introduction

In the field of biomaterials and mechano-biology, the regulation of cellular functions using the properties of culture substrates, such as substrate stiffness [1,2] or nanotopography [3,4] of the adhesion surface, has been attracting attention. For instance, it has been observed that a variation in matrix stiffness from soft to rigid can direct mesenchymal stem cell (MSC) fate [5]. Apart from this, we have developed cell culture surfaces using supramolecular polymers, polyrotaxanes (PRXs), and succeeded in regulating cellular functions such as adhesion [6], proliferation [7], and differentiation [8]. PRX is a supermolecule consisting of cyclic molecules threaded onto the axis polymer. There are many combinations of axis polymers and cyclic molecules, for example, polyethylene glycol (PEG) and α-cyclodextrin (α-CD) [9]. The structural features of PRXs include mechanical interlocking of cyclic molecules with a linear polymer. Consequently, PRXs exhibit unique properties of molecular mobility such as the sliding and rotating of cyclic molecules along the polymer chain. The molecular mobility of PRX surfaces can be modulated by changing the number of threaded α-CDs and modifying functional groups onto the α-CDs [10].

By adjusting the molecular mobility, we succeeded in regulating the morphology [11] and differentiation of the MSCs [12]. For example, when the MSCs were cultured on PRX surfaces with low mobility, they differentiated predominantly into osteoblasts. On the other hand, when cultured on PRX surfaces with high mobility, they preferentially differentiated into adipocytes. PRX surfaces with low mobility had a higher level of the Ras homolog gene family, member A (RhoA) gene expression, and Rho-associated coiled-coil-containing protein kinase (ROCK) activity in adherent MSCs, leading to the formation of mature actin stress fibers and MSC differentiation into osteogenic cells [12]. These findings strongly suggest that the molecular mobility of PRXs may be useful as a structural feature for regulating cellular responses.

However, it is not clear how PRX surfaces differ from commercially available culture surfaces. The effectiveness and advantages of the PRX surfaces as culture environments may be revealed by comparison with commercially available surfaces. As a commercially available culture surface, a tissue culture polystyrene (TCPS) surface, which is the gold standard for cell culture surfaces, can be used.

In the present study, we analyzed the cellular responses to the surface of a PRX or TCPS using various cell types. In particular, fibroblast, preosteoblast, and preadipocyte cell lines were selected, since these cells are known to alter proliferation and differentiation depending on the properties of the cell culture surfaces [13,14,15]. In order to evaluate cellular proliferation, we performed a comprehensive gene expression analysis on the proliferative ability of each cell. We also examined the differentiation ability of preosteoblasts and preadipocytes.

## 2. Materials and Methods

### 2.1. Materials

Dulbecco’s Modified Eagle Medium (DMEM), α-Minimum Essential Medium (α-MEM), 100 U/mL penicillin, 100 mg/mL streptomycin, trypsin/ethylenediaminetetraacetic acid (EDTA) solution, phosphate buffered saline (PBS), 99% ethanol, 4% paraformaldehyde, 2-propanol, alizarin red S, oil red O, L-ascorbic acid phosphate magnesium salt, dexamethasone, and dimethyl sulfoxide (DMSO) were purchased from FUJIFILM Wako Pure Chemical Corporation (Osaka, Japan). Fetal bovine serum (FBS) was obtained from Gibco BRL, Life Technologies (Carlsbad, CA, USA). Disodium β-glycerophosphate pentahydrate and digitonin was purchased from Tokyo Chemical Industry (Tokyo, Japan). 3-Isobutyl-1-methylxanthine (IBMX) was obtained from Nacalai Tesque (Kyoto, Japan). A 24-well tissue culture polystyrene (TCPS) plate was purchased from Thermo Fisher Scientific (Waltham, MA, USA). MC3T3-E1 subclone 4 (mouse preosteoblast cell line) and BALB/3T3 clone A31 (mouse fibroblast cell line) were purchased from the American Type Culture Collection (Manassas, VA, USA). The MC3T3-G2/PA6 (mouse preadipocyte cell line) was provided by RIKEN Cell Bank (Tsukuba Science City, Japan).

Methylated PRX triblock copolymers (Me-PRX), consisting of methyl group-modified α-CDs threaded onto a PEG chain (*M*_n_ = 20,000) as a middle PRX segment and poly(benzyl methacrylate) (*M*_n_ = 54,400) at both terminals of the PEG as anchoring segments, were synthesized and used as previously reported [16]. The molecular weight (*M*_n_) of Me-PRX, with an α-CD threading number of 89.8, was 108,200. The number of modified methyl groups in the threaded α-CD was 15.

### 2.2. Preparation of the Me-PRX Surfaces

**Figure 1:**
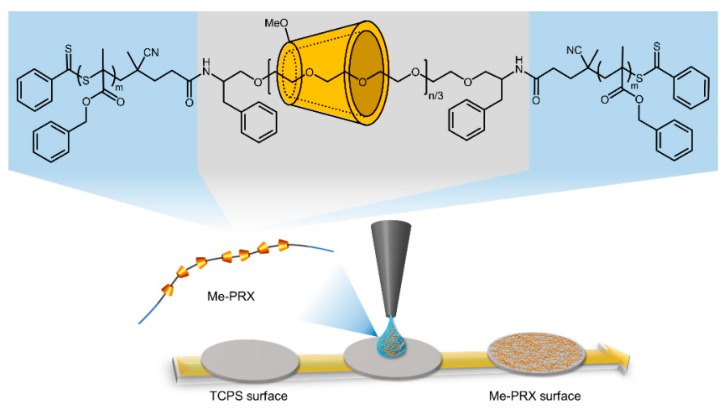
Chemical structure of methylated polyrotaxane triblock copolymer (Me-PRX) and preparation of Me-PRX surfaces.

Me-PRX was dissolved in DMSO to prepare 0.05% polymer solutions (Figure 1). Next, 30 μL of solution was spread on the 24-well TCPS surface and dried at 60 °C for 18 h to obtain Me-PRX surfaces (Figure 1). All Me-PRX surfaces were sterilized by ultraviolet irradiation for 20 min on a clean bench and washed three times with 500 μL of PBS prior to the cell experiments.

### 2.3. Morphology and Proliferation of the Cells

In order to evaluate the morphology of adhering cells on surfaces, BALB/3T3, MC3T3-E1, and MC3T3-G2/PA6 cells were seeded on Me-PRX and TCPS surfaces at a density of 2.0 × 10^3^ cells/cm^2^ and cultured using standard media (for BALB/3T3 cells: DMEM, 10% FBS, 100 U/mL penicillin, 100 mg/mL streptomycin; for MC3T3-E1 and MC3T3-G2/PA6 cells: α-MEM, 10% FBS, 100 U/mL penicillin, 100 mg/mL streptomycin) at 37 °C in a humidified atmosphere with 5% CO_2_ for 24 h. Adherent cells were fixed in 4% paraformaldehyde at room temperature for 10 min, washed with PBS, and permeabilized with 50μg/mL digitonin for 5 min. For actin filament staining in adherent cells, the cells were incubated with Alexa Fluor 555 phalloidin (1:100) (Invitrogen, Carlsbad, CA, USA) in PBS at room temperature for 30 min and washed with PBS. Nuclear DNA was stained with Hoechst 33342 (Dojindo, Kumamoto, Japan) (1:500). After the cells were washed with PBS, fluorescent images of the stained cells were acquired with a fluorescence microscope (IX71, Olympus, Tokyo, Japan) using CellSens standard software (Olympus). The area and aspect ratio of the spreading cells were analyzed using ImageJ (NIH, Bethesda, MD, USA). The aspect ratio was determined by approximating the cell shape to an ellipse and dividing the long axis by the short axis. At least 50 cells from each surface were analyzed. In order to evaluate the proliferation of adhering cells on surfaces, each cell was cultured in a standard growth medium for 5 days. The culture medium was changed every 3 days. The cellular density of BALB/3T3, MC3T3-E1, MC3T3-G2/PA6 cells on each surface was determined by counting the cells from the captured images at 1 d intervals over 5 d of cultivation. The doubling time of each cell was calculated from the change in the number of adherent cells from 48 h to 96 h. The adherent cells were observed using a phase contrast microscope (IX71; Olympus) equipped with a dual CCD digital camera (DP80; Olympus).

### 2.4. DNA Microarray Analysis

**Figure 2:**
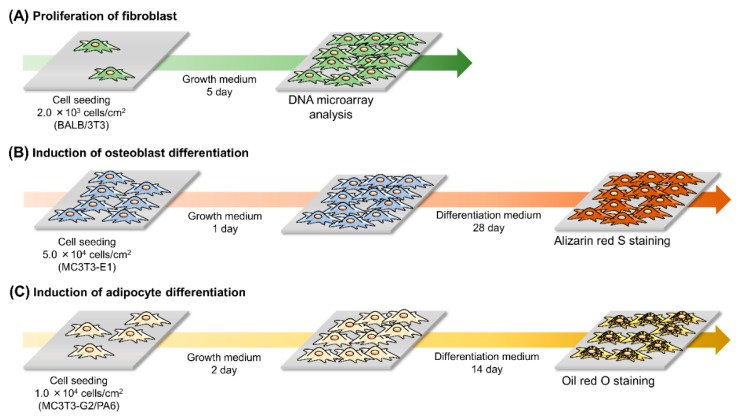
Experimental design for (**A**) fibroblast proliferation, (**B**) induction of osteoblast differentiation, and (**C**) induction of adipocyte differentiation.

To perform comprehensive gene expression analysis (Figure 2), BALB/3T3 cells were seeded on Me-PRX and TCPS surfaces at a density of 2.0 × 10^3^ cells/cm^2^ and cultured with standard growth media (DMEM, 10% FBS, 100 U/mL penicillin, and 100 mg/mL streptomycin) at 37 °C in a humidified atmosphere with 5% CO_2_. The culture medium was changed every 3 days. After 5 days, the total RNA was extracted from cells using the FastGene^TM^ RNA Premium Kit (NIPPON Genetics, Tokyo, Japan). After verifying RNA quality, cDNA was synthesized and purified using the Gene Chip^TM^ WT PLUS Reagent Kit (Thermo Fisher Scientific, Waltham, MA, USA). Next, in vitro transcription and T7 RNA amplification were performed. Fragmentation and labeling of cDNA were performed using a GeneChip^TM^ Hybridization, Wash, and Stain Kit (Thermo Fisher Scientific). The prepared samples were hybridized, washed, and stained using an automated system (GeneChip^TM^ Scanner 3000 7G system; Thermo Fisher Scientific). DNA microarray experiments were performed using the Clariom^TM^ S Assay Mouse (Thermo Fisher Scientific). The hybridization signal on the chip was scanned using a GeneChip 3000 7G scanner (Thermo Fisher Scientific) and processed by a microarray data analysis tool in consideration of the National Center for Biotechnology Information (NCBI) database and analyzed by software from Filgen Inc., Nagoya, Japan. The DNA microarray expression profiles were compared between cells on Me-PRX surfaces and cells on TCPS surfaces.

### 2.5. Differentiation of the Cells

For induction of osteogenic differentiation, MC3T3-E1 cells were seeded on Me-PRX and TCPS surfaces at a density of 5.0 × 10^4^ cells/cm^2^ and cultured using a standard growth medium at 37 °C in a humidified atmosphere with 5% CO_2_ (Figure 2). After 24 h of incubation, the growth medium was replaced with an osteoblast differentiation medium (α-MEM, 10% FBS, 100 U/mL penicillin, 100 mg/mL streptomycin, 50 μg/mL L-ascorbic acid phosphate magnesium salt, and 10 mM disodium β-glycerophosphate pentahydrate). The medium was changed every 3 days. After culturing MC3T3-E1 cells in osteoblast differentiation medium for 28 days, the adherent cells were washed with PBS and fixed with 99% ethanol for 10 min at room temperature. To evaluate mineralization, alizarin red S staining was performed. Briefly, fixed cells were washed twice with Milli-Q water and stained with 1% alizarin red S solution for 10 min at room temperature. Next, the stained cells were washed five times with Milli-Q water. Images of cells were acquired using a phase contrast microscope (IX71; Olympus) equipped with a dual CCD digital camera (DP80; Olympus). Stained areas of the images were quantified using ImageJ software (NIH, Bethesda, MD, USA).

To induce adipogenic differentiation, MC3T3-G2/PA6 cells were seeded on Me-PRX and TCPS surfaces at a density of 1.0 × 10^4^ cells/cm^2^ and cultured using a standard growth medium at 37 °C in a humidified atmosphere with 5% CO_2_ (Figure 2). After 48 h incubation, the growth medium was replaced with an adipocyte differentiation medium (α-MEM, 10% FBS, 100 U/mL penicillin, 100 mg/mL streptomycin, 0.5 mM IBMX, and 0.25 mM dexamethasone). The medium was replaced every 3 days. After culturing MC3T3-G2/PA6 cells in adipocyte differentiation medium for 14 days, the adherent cells were washed with PBS and fixed with 4% paraformaldehyde for 10 min at room temperature. To evaluate adipogenesis, Oil Red O staining was performed. Oil red O solution in 60% 2-propanol was added to each well. The plates were incubated at room temperature for 20 min. Next, the stained cells were washed twice with PBS. Images of cells were acquired using a phase contrast microscope (IX71; Olympus) equipped with a dual CCD digital camera (DP80; Olympus). Stained areas of the images were quantified using ImageJ software.

### 2.6. Statistical Analysis

The data were analyzed using Student’s *t*-test. All data were presented as the mean ± standard deviation (S.D.).

## 3. Results and Discussion

### 3.1. Morphology of the Cells

**Figure 3:**
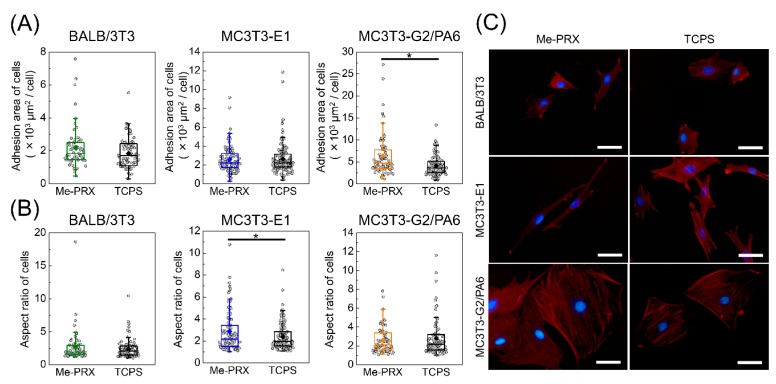
(**A**) The adhesion area of BALB/3T3, MC3T3-E1, and MC3T3-G2/PA6 cells on the surface of Me-PRX and tissue culture polystyrene (TCPS) surfaces. (**B**) Aspect ratio of cells on each surface. Each rectangle represents the mean. Data are presented as mean ± S.D., n ≥ 50. *p < 0.05 (Student’s *t*-test). (**C**) Fluorescent images of cells on each surface after 24 h culture. Blue, nucleus; red, actin filament. Scale bars, 50 μm.

We investigated the difference in cell spreading between Me-PRX and TCPS surfaces in terms of areas of the cells. The adhesion areas of BALB/3T3, MC3T3-E1, and MC3T3-G2/PA6 cells on Me-PRX surfaces were 2190 ± 1310, 2570 ± 1440, and 6370 ± 4850 μm^2^, respectively. The adhesion areas of BALB/3T3, MC3T3-E1, and MC3T3-G2/PA6 cells on TCPS surfaces were 1850 ± 900, 2680 ± 1640, and 4170 ± 2300 μm^2^, respectively (Figure 3A,C). MC3T3-G2/PA6 cells on Me-PRX surfaces had significantly wider spreading than on TCPS surfaces. At the same time, the aspect ratios of BALB/3T3, MC3T3-E1, and MC3T3-G2/PA6 cells on Me-PRX surfaces were 2.7 ± 2.5, 2.9 ± 1.8, and 2.6 ± 1.4, respectively. The aspect ratios of BALB/3T3, MC3T3-E1, and MC3T3-G2/PA6 cells on TCPS surfaces were 2.4 ± 1.4, 2.3 ± 1.1, and 2.8 ± 1.9, respectively (Figure 3B,C). The aspect ratio of MC3T3-E1 cells on Me-PRX surfaces was significantly larger than that on TCPS surfaces. 

Cell spreading and morphology play an important role in cellular functions and are known to affect the activation of intracellular signaling pathways related to cellular proliferation and differentiation [17,18]. Chen and co-workers have reported that the morphology and spreading regulate commitment of human MSCs to adipocyte or osteoblast differentiation via RhoA/ROCK signaling pathways [19]. In addition, the RhoA/ROCK signal pathway also regulates the cellular proliferation [20]. It is expected that PRX surfaces may differ in cellular proliferation and differentiation from TCPS surfaces. Subsequently, both proliferation and differentiation of each cell type on the Me-PRX and TCPS surfaces were evaluated.

### 3.2. Proliferation of the Cells

**Figure 4:**
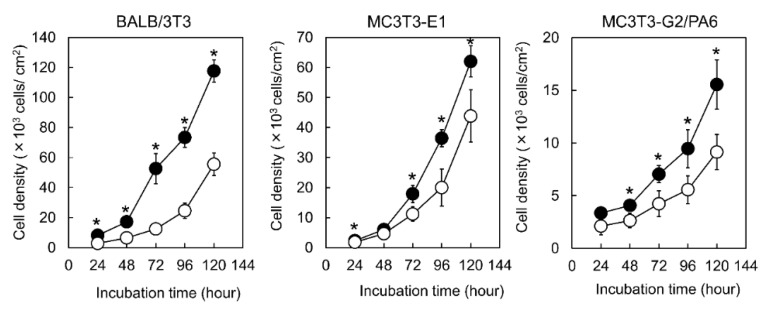
Growth curves of BALB/3T3, MC3T3-E1, and MC3T3-G2/PA6 cells on the surface of Me-PRX (closed circles) and TCPS (open circles) during a 5-day cultivation period. The initial cell seeding density was 2.0 × 10^3^ cells/cm^2^. Data are presented as mean ± standard deviation (S.D.), n = 4. *p < 0.05 (Student’s *t*-test).

To evaluate proliferation of BALB/3T3, MC3T3-E1, and MC3T3-G2/PA6 cells, the number of adherent cells on Me-PRX and TCPS surfaces was counted every 24 h (Figure 4). Adherent cells on Me-PRX surfaces were significantly larger than on TCPS surfaces, regardless of the cell type. The doubling times of BALB/3T3, MC3T3-E1, and MC3T3-G2/PA6 cells on Me-PRX surfaces were 23.0 ± 1.6, 18.5 ± 0.7, and 40.7 ± 6.2, respectively. The doubling times of BALB/3T3, MC3T3-E1, and MC3T3-G2/PA6 cells on TCPS surfaces were 25.3 ± 4.0, 24.5 ± 8.2, and 44.6 ± 4.3, respectively. The doubling times of cells on Me-PRX surfaces tended to be shorter compared to the TCPS surfaces.

**Figure 5:**
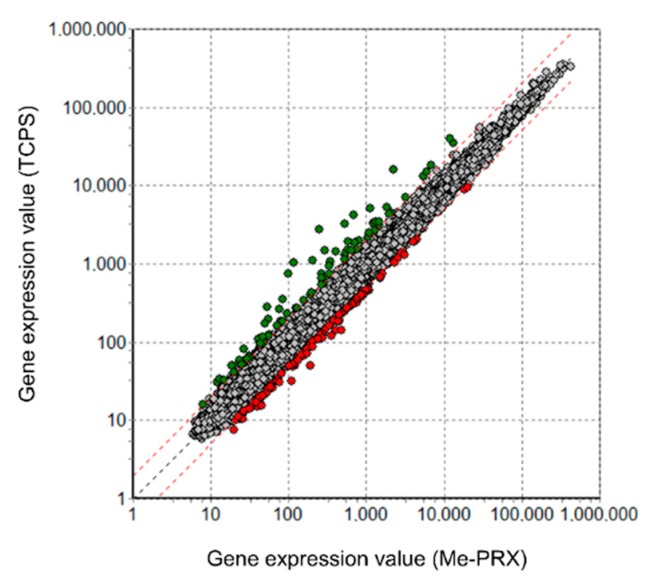
Scatter plot representation of global gene expression profiles in BALB/3T3 cells on Me-PRX (twofold change threshold). Global gene expression profiles of cells on Me-PRX surfaces were compared with those of cells on TCPS surfaces. Red circles: upregulated genes; green circles: downregulated genes.

To determine how the Me-PRX surfaces affect proliferation of the adhering cells, DNA microarray analysis was performed using BALB/3T3 cells. We compared the gene expression profiles of the cells cultured on Me-PRX surfaces and on TCPS surfaces. A fold change cutoff of 2 for upregulation of genes and a p-value cutoff of 0.05 were set to identify the genes to be analyzed. Our results indicated that out of 28,846 genes, at a twofold change cutoff, 205 genes were differentially expressed (Figure 5). Cells cultured on Me-PRX surfaces had increased expression of 121 genes and decreased expression of 84 genes. 

The differentially expressed genes were subjected to pathway analysis based on the NCBI database, and the significant pathways of upregulated genes are listed in Table 1. Even though no additional factors such as bioactive molecules and growth factors were supplied to the culture system, it was found that several pathways were activated by the Me-PRX surface. Especially, the pathways related to integrin genes were activated. It has been known that integrins are representative proteins involved in cellular adhesion, and Me-PRX surfaces have shown significant activation of integrin-mediated cell adhesion pathway and focal adhesion pathway in the adhering cells. In addition, the expression of integrins alpha and beta, located upstream of these pathways [21,22], was increased, as well as the expression of Map2k6, a component of the extracellular signal-regulated kinase (ERK) signaling related to cell proliferation [23]. There was no discrepancy between the significant increase in upstream gene expression in these pathways and the upregulation of cellular proliferation located downstream of the pathway. It is possible that Me-PRX surfaces provided a mechanical cue necessary for the activation of these pathways. This time, we identified only a portion of the gene expression profile; however, in future experiments, by analyzing gene expression over time, we can report on observed changes in downstream gene expression as well.

### 3.3. Differentiation of the Cells

**Figure 6:**
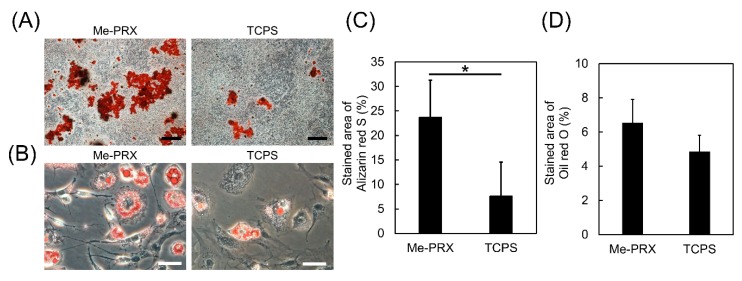
(**A**) Alizarin red S staining images of MC3T3-E1 cells on Me-PRX and TCPS surfaces after a 4-week incubation in osteogenic differentiation media. Scale bar: 500 μm. (**B**) Oil red O staining images of MC3T3-G2/PA6 cells on each surface after a 2-week incubation in adipogenic differentiation media. Scale bar: 50 μm. (**C**) Relative area of the alizarin red S stained cultures was analyzed using Image J software. (**D**) Relative area of Oil Red O stained cells was analyzed using Image J software. Data are presented as mean ± S.D., n = 6. *p < 0.01 (Student’s *t*-test).

To evaluate cellular differentiation, MC3T3-E1 cells were cultured in an osteoblast differentiation medium, while MC3T3-G2/PA6 cells were cultured in an adipocyte differentiation medium. To confirm formation of mineralized nodules, MC3T3-E1 cultures were stained with Alizarin Red S. The mineralized area on Me-PRX surfaces was significantly larger compared to TCPS (Figure 6A,C). Kilian et al. reported that an increase in the cell spreading enhanced proliferation and osteogenesis during the MSCs’ culture [24]. Therefore, we expected that Me-PRX surfaces would promote osteoblast differentiation. MC3T3-G2/PA6 cells were stained with Oil Red O. There was no significant difference in oil red O staining between the two surfaces (Figure 6B,D). 

## 4. Conclusions

In this study, Me-PRX surfaces were found to significantly promote proliferation of fibroblast, preosteoblast, and preadipocyte cell lines compared to TCPS surfaces. DNA microarray results suggested that Me-PRX surfaces activated integrin-mediated cell adhesion and focal adhesion. In addition, Me-PRX surfaces effectively enhanced osteoblast differentiation from the preosteoblasts. The structural features of PRX surfaces may act as mechanical cues to stimulate cell proliferation and osteoblast differentiation. Although we have yet to elucidate this detailed mechanism, PRX surfaces with dynamic features may provide a suitable environment for cells in vitro. Furthermore, the promotion of cellular proliferation and differentiation without bioactive molecules such as growth factors is a great advantage for implantable scaffold applications in tissue regeneration. These findings could contribute important concepts to the design of biomaterials used in regenerative medicine.

## Figures and Tables

**Table 1:** Pathways upregulated on the Me-PRX surfaces.

Name of Pathway	Changed Genes	Total Genes	Z Score	*p*-Value	Gene Symbols
Primary Focal Segmental Glomerulosclerosis	4	72	5.803	0.0006	Itga3, Itgb3, kirrel3, Tgfb1
ACE Inhibitor Pathway	2	8	9.435	0.0012	Bdkrb2, Kng1
Integrin-mediated Cell Adhesion	4	100	4.718	0.002	Itga3, Itga6, Itgb3, Map2k6
Toll Like Receptor Signaling	2	33	4.325	0.0147	Map2k6, Traf3
Spinal Cord Injury	3	97	3.428	0.0156	Selp, Gdnf, Tgfb1
Focal Adhesion	4	184	3.021	0.0167	Itga3, Itga6, Itgb3, Map2k6
Endochondral Ossification	2	62	2.884	0.0456	Tgfb1, Sox5
